# Which hematological markers have predictive value as early indicators of severe COVID-19 cases in the emergency department?

**DOI:** 10.3906/sag-2008-6

**Published:** 2021-03-15

**Authors:** İshak ŞAN, Emin GEMCİOĞLU, Mehmet DAVUTOĞLU, Ramis ÇATALBAŞ, Berkan KARABUĞA, Enes KAPTAN, Abdulsamet ERDEN, Orhan KÜÇÜKŞAHİN, İhsan ATEŞ, Selma KARAAHMETOĞLU, İmran HASANOĞLU, Osman İNAN, Büşra Nur ÜNAL, Ecem ERDEMİR, Fatih Ahmet KAHRAMAN, Rahmet GÜNER

**Affiliations:** 1 Department of Emergency, Faculty of Medicine, University of Health Sciences, Ankara, Turkey; 2 Department of Internal Medicine, Ankara City Hospital, Ankara, Turkey; 3 Department of Internal Medicine, Faculty of Medicine, Yıldırım Beyazıt University, Ankara, Turkey; 4 Division of Rheumatology, Department of Internal Medicine, Ankara City Hospital, Ankara, Turkey; 5 Division of Rheumatology, Department of Internal Medicine, Faculty of Medicine, Yıldırım Beyazıt University, Ankara, Turkey; 6 Department of Infectious Diseases and Clinical Microbiology, Faculty of Medicine, Yıldırım Beyazıt University, Ankara, Turkey; 7 Department of Emergency, Faculty of Medicine, Yıldırım Beyazıt University, Ankara, Turkey

**Keywords:** COVID-19, SARS-CoV-2, hematological tests, predictive values of tests

## Abstract

**Background/aim:**

Coronavirus 2019 disease (COVID-19), caused by severe acute respiratory syndrome coronavirus 2 (SARS-CoV-2), is a pandemic infectious disease that causes morbidity and mortality. As a result of high mortality rate among the severe COVID-19 patients, the early detection of the disease stage and early effective interventions are very important in reducing mortality. Hence, it is important to differentiate severe and nonsevere cases from each other. To date, there are no proven diagnostic or prognostic parameters that can be used in this manner. Due to the expensive and not easily accessible tests that are performed for COVID-19, researchers are investigating some parameters that can be easily used. In some recent studies, hematological parameters have been evaluated to see if they can be used as predictive parameters.

**Materials and methods:**

In the current study, almost all hematological parameters were used, including the neutrophil/lymphocyte ratio, platelet/lymphocyte ratio, monocyte/lymphocyte ratio, mean platelet volume to lymphocyte ratio, mean platelet volume to platelet ratio, plateletcrit, and D-dimer/fibrinogen ratio, neutrophil/lymphocyte/platelet scoring system, and systemic immune-inflammation index. A total of 750 patients, who were admitted to Ankara City Hospital due to COVID-19, were evaluated in this study. The patients were classified into 2 groups according to their diagnosis (confirmed or probable) and into 2 groups according to the stage of the disease (nonsevere or severe).

**Results:**

The values of the combinations of inflammatory markers and other hematological parameters in all of the patients with severe COVID-19 were calculated, and the predicted values of these parameters were compared. According to results of the study, nearly all of the hematological parameters could be used as potential diagnostic biomarkers for subsequent analysis, because the area under the curve (AUC) was higher than 0.50, especially for the DFR and NLR, which had the highest AUC among the parameters.

**Conclusion:**

Our findings indicate that, the parameters those enhanced from complete blood count, which is a simple laboratory test, can help to identify and classify COVID-19 patients into non-severe to severe groups.

## 1. Introduction

Coronavirus 2019 disease (COVID-19), caused by severe acute respiratory syndrome coronavirus 2 (SARS-CoV-2), is a pandemic infectious disease that causes morbidity and mortality. To date there are no proven diagnostic or prognostic parameters, but clinicians can use predictive parameters, such as the leukocyte and lymphocyte counts,

C-reactive protein (CRP), D-dimer, and ferritin levels, and radiological imaging [1–3]. In the inflammation process of COVID-19, CRP, platelet, ferritin, and leukocyte values may increase, while albumin and lymphocyte values may decrease [4–6].

White blood cells (WBCs), including neutrophils, lymphocytes, monocytes, have been used as inflammatory biomarkers in infections, autoinflammatory diseases, and cancers that contain inflammatory processes, and are the most generally performed laboratory tests [5–8].

The neutrophil/lymphocyte ratio (NLR) is a strong biomarker, indicating acute and chronic inflammatory status, is reliable, cost-effective, and easily applicable, and is considered as a stronger inflammatory marker than the individual assessment of the neutrophil count or lymphocyte count [7–10]. The platelet/lymphocyte ratio (PLR), monocyte/lymphocyte ratio (MLR), mean platelet volume/lymphocyte ratio (MPVLR), mean platelet volume/platelet ratio (MPVPR) are the parameters that are easily calculated from the complete blood count. Earlier studies have shown that these parameters have prognostic value for various diseases, in addition to the early recognition of infection and inflammation [3,11,12].

One of the newly introduced parameters to measure the degree of inflammation is the systemic immune-inflammation index (SII). This index is obtained by multiplying the platelet count by the NLR [13–15]. The SII has been accepted as an indicator of inflammatory status, with the added feature of being a prognostic marker in malignancy [3,13–15].

Scoring systems with predictive value that have been developed using hematological parameters are also important. An example of this is the neutrophil/ lymphocyte/platelet scoring system (NLP score). Studies using this scoring system found that COVID-19 patients with an NLP score greater than 6 had a high risk of severe disease [10].

D-dimer is a fibrin degradation product and has been specifically associated with secondary fibrinolysis. However, fibrinogen is a coagulation product synthesized from the liver, and it is also an acute phase reactant. In previous studies, the D-dimer/fibrinogen ratio (DFR) was found to be significantly correlated with inflammation, malignancy, and thromboembolic events [16–20]. The mechanism responsible for the inclusion of hematological parameters in this process is the effects of cytokines and chemokines released from neutrophils, monocytes, and macrophages migrating to the inflammation site, in addition, the fact that the reactive oxygen species, caused by lysosomal enzymes released from neutrophils, cause the immature, young erythrocytes and platelets to enter peripheral circulation from the bone marrow [7].

Hematological parameters are important to support the diagnosis of COVID-19. The aim in this study was to evaluate the relationship of hematological parameters (NLR, PLR, MLR, MPVLR, DFR, MPVPR, plateletcrit, NLP score, and SII) with confirmed and probable COVID-19 cases. In addition, it was aimed to evaluate whether these biomarkers predicted the severity of the disease with the first-look values in the emergency department (ED) in COVID-19 patients. There are studies that have evaluated whether one or more hematological parameters can predict severe disease in COVID-19, but the current study was the first in which almost all of the hematological parameters were evaluated together.

## 2. Materials and methods

This study was conducted at the Internal Medicine and Infectious Diseases wards of Ankara City Hospital due to COVID-19, and the patients were evaluated retrospectively. Patients younger than 18 years of age, those with active malignancy, and pregnant women were excluded from the study. Ethical approval for the study was obtained from the Ethics Committee of Ankara City Hospital (approval number: E1-20-999). The age, sex, comorbidity, and medications of the patients, as well as the D-dimer, fibrinogen, complete blood count, biochemical parameters, CRP, sedimentation, and thorax computerized tomography (CT) findings on the ED admission were recorded. Demographic, clinical, laboratory, imaging examination, treatment, and outcome data were collected using a standardized case-report form. All data were checked by 2 physicians (MD and RC), and then a third researcher (BK) determined any differences in interpretation between the 2 primary reviewers.

To understand the relation with disease severity, 9 inflammatory markers were used. Moreover, 9 inflammatory factors, including the NLR, PLR, MLR, MPVLR, MPVPR, plateletcrit, DFR, NLP, and SII (platelet × neutrophil/lymphocyte) were used in this analysis.

From all of the patients, nasal and/or pharyngeal swab specimens were collected, and reverse-transcriptase polymerase chain reaction (RT-PCR) assays were performed. The diagnosis of COVID-19 was based on positivity of the real-time RT-PCR results.

According to the diagnosis, the patients were classified into 2 groups, as confirmed and probable COVID-19 (Figure). According to the stage of the disease, the first group comprised non-severe patients who had any of the following: slight symptoms, fever, respiratory tract symptoms, and no radiological findings or pneumonia findings on radiological examination. The second group comprised severe patients who had any of the following: tachypnea with a respiration rate >30 beats/min, resting oxygen saturation < 92%, arterial partial oxygen pressure (PaO_2_)/fraction of inspired oxygen (FiO_2_) < 301 mmHg, radiological aggravation greater than 50% within 24–48 h, respiratory failure and mechanical ventilation, shock, and organ failure requiring intensive care unit admission. To determine severe patients on admission to the hospital for a respiratory illness, the slightly modified and adopted interim guidance of the World Health Organization^1^ was used [21]. The outcome of the follow-up was the occurrence of severe illness, and the end of follow-up time was 1st of June 2020.

Hospitalization, treatment, management, and discharge decisions of the patients were made according to the guidelines of the Turkish Ministry of Health^2^.

## 3. Statistical analysis

The data were analyzed using IBM SPSS for Windows v: 25.0 (IBM Corp., Armonk, NY, USA) and MedCalc 15.8 (Franz Faul, Universitat Kiel, Germany). While the frequency, percentage, mean, standard deviation, median, and IQR were used as descriptive statistical methods, the chi square (c2) test was used to compare the qualitative data. The consistency of the data to normal distribution was evaluated using the Kolmogorov–Smirnov and Shapiro–Wilk tests. The Mann–Whitney U test was used to compare the nonnormally distributed data. While the receiver operating characteristic (ROC) curve method was used to determine the discrimination of the variables, binary logistic regression was used to determine the risk rates. Statistical significance was accepted as p < 0.05.

## 4. Results

Of the 750 patients included in the study, 388 (51.7%) were confirmed COVID-19 patients, while 362 (48.3%) were probable COVID-19 patients. Of these 750 patients, 442 (58.9%) were males. The median (IQR) age of all of the patients was 49 (28) years (Table 1). The median (IQR) age in the probable group was 55 (27) years, while it was 45 (25) years in the confirmed group (p < 0.0001).

The frequency of cough, fever, myalgia, anosmia, ageusia, and arthralgia was significantly higher in the confirmed group than in the probable group (Table 1). The frequency of dyspnea was significantly higher in the probable group than in the confirmed group. There was no statistically significant difference between the probable and confirmed groups in terms of headache, nausea, diarrhea, back pain, or abdominal pain.

The frequency of smoking and any comorbidity was significantly higher in the probable group than in the confirmed group (p = 0.008 and p < 0.0001). While the need for intensive care was higher in the probable group (p < 0.0001), mortality was higher in the confirmed group (p < 0.0001).

Considering the indices, the MPV/lymphocyte ratio and the MPV/platelet ratio were significantly higher in the confirmed group than in the probable group (p < 0.0001) (Table 2). The NLR and SII scores were significantly higher in the probable group than in the confirmed group (Table 2).

Detailed comparison of hematological parameters in terms of severity vs nonseverity in all 3 groups (all patients, confirmed group, and probable group) is shown in Table 3. Severe disease patients in all 3 groups (all patients, confirmed group, and probable group) were older than the non-severe patients (p < 0.0001). In all 3 groups, severe disease patients had more comorbidities than the non-severe patients (p < 0.0001). Except for 1 (MPV/platelet ratio) of the 8 indices (NLR, excluding the PLR, MLR, MPV/lymphocyte, MPV/platelet ratio, and DFR, and SII and NLP scores), all of the indices showed a statistically significant differences in terms of severity of the disease between the 3 groups (Table 3).

The multivariate logistic regression model for severe disease consisted of the variables, including age, male sex, plateletcrit, NLR, PLR, MLR, MPV/lymphocyte, MPV/ platelet, and DFR, and SII and NLP scores which are given in Table 4. In the multivariate logistic regression analyses, DFR in the highest tertile (HR: 1.206, 95% CI: 1.049–1.387, p = 0.009) was determined as an independent predictor of severe disease in COVID-19. In the multivariate analyses, the serum plateletcrit value, NLR, PLR, and age were found to be an independent predictor of severe disease in COVID-19.

The values of 8 combinations of inflammatory markers and other hematological parameters in all of the patients with severe COVID-19 were calculated, and the predicted values of these parameters were compared in the ROC analysis. In Table 5, area under the curve (AUC) of the DFR, NLR, and SII were 0.767, 0.750, and 0.740, respectively. The optimal cut-off values were >0.22, >3.59, and >998.28 for the DFR, NLR, and SII, respectively. Nearly all of the hematological parameters could be used as potential diagnostic biomarkers for subsequent analysis because their AUC was higher than 0.50.

## 5. Discussion

Laboratory medicine has a crucial role in the diagnosis and management of variable diseases [22]. Recent studies have reported the routine blood test results of COVID-19 patients and shown the differences between the nonsevere and severe COVID-19 patients [21-23-24]. Subtypes of the WBCs alone are good predictors of inflammation but the NLR is superior to them because it combines the value of 2 subtypes [8]. For COVID-19, it is known that the lymphocyte count decreases, thus the NLR becomes valuable in this situation. Inflammatory storm and severity of COVID-19 have a close relation [25], and as a result, prognosis of the disease can be better reflected using the NLR. Among patients diagnosed with COVID-19, it was found that patients with severe symptoms had a higher NLR than patients with mild-to-moderate symptoms. High NLR levels promote COVID-19 progression. According to this study, an increased NLR on admission was accepted as an independent risk factor for severe cases of COVID-19. In a meta-analysis, it was shown that the neutrophil count and NLR were positively correlated and the lymphocyte count was negatively correlated to the severity of COVID-19 [26]. Hence, NLR is a significant and valuable parameter in determining the severity of the disease.

Platelets, i.e. the lymphocyte count, are also correlated with inflammation. In COVID-19 the lower lymphocyte count was correlated with an increased risk of inflammation [27].

PLR has been recently used to predict prognosis of thrombotic and inflammatory diseases. Since COVID-19 has an inflammatory process, and in many cases, thrombotic events have been reported, PLR was thought to have the potential to show the prognosis of COVID-19 [28]. Some recent studies have shown higher PLR levels in severe COVID-19 patients when compared to non-severe patients [29,30]. In the current study, it was found that the PLR value measured on admission increased in parallel with the progression of severity. Hence, increased PLR on admission was accepted as an independent risk factor for severe cases of COVID-19 in this study.

As for the other WBC subtypes, the monocyte count is also related with inflammation and is expected to be elevated [31]. Recently, some new indicators of disease severity have been used, such as the MLR, and they were related with various diseases like rheumatic disease and cancer [7–32]. In one study, the MLR was demonstrated as to be the best marker of infection in cirrhotic patients [33]. In another study, among COVID-19 patients who showed progression on chest CT scan, the MLR was dramatically higher when compared to the other markers, such as the aspartate aminotransferase-lymphocyte ratio index, aspartate aminotransferase-platelet ratio index, NLR, PLR, and SII [34]. Similarly, in the current study, the MLR was significantly higher in the severe disease patient group than in the non-severe patient group, but the MLR on admission was not accepted as an independent risk factor for severe cases of COVID-19.

Platelet function and activation is reflected by the MPV, and it could be used as a marker for inflammation [35]. The MPV is also a valuable marker in systemic inflammatory diseases and is thought to have a parallel correlation with CRP [36]. MPV levels have shown depressed levels during systemic inflammatory situations [37]. The mechanism for this is still unclear, but during the inflammatory process, a defect in thrombopoiesis could be responsible for this situation [12].

In another study, it was reported that the MPVLR could be used as a new marker for inflammatory diseases and thrombotic events [38,39].

The data obtained herein from the COVID-19 patients were similar to those of previous studies, and it is suggested that the MPVLR can be used as a new parameter to determine disease severity groups. However, it is not an independent risk factor for severe cases of COVID-19, according to the results of the current study.

Hypercoagulability is a common finding among severe COVID-19 cases [40]. This is the main reason why thromboembolic events have been seen so frequently in severe COVID-19 patients [41,42]. As other blood cell platelets are also affected by core inflammatory cytokines, such as interleukin (IL)-1, IL-6, INF-γ, and tumor necrosis factor, they have a close relationship with inflammation, and that is why they were accepted herein as acute phase reactant [43]. In some studies, MPVPR levels have been foundtobehigherinvariouspatientswhohadinflammatory process like sepsis and pancreatitis. Additionally, increased thrombosis and mortality were reported in Behçet disease [44–46]. Among the English literature on the MPVPCR in COVID-19, the current study is unique with its findings. Contrary to other studies, MPVPCR was not found to be a significant marker to classify COVID-19 patients into severe or nonsevere groups.

In blood, the percentage of platelet volume is defined by the plateletcrit. Recent studies have reported that plateletcrit may be a more effective marker compared to MPV [11,47,48]. The plateletcrit has been shown to be increased and associated with CRP and d-dimer levels in active inflammation [49].

The plateletcrit was significantly higher in the severe group when compared to all of the patients, as severe vs. non-severe. It is an independent risk factor for severe cases of COVID-19 according to the current study. Considering the studies showing that plateletcrit increases in active inflammation, the results of the present study were not surprising.

Fibrinogen levels increase as an acute phase reactant in various diseases that have inflammatory processes, such as hemodynamic impairments, cardiac-lung-aortic diseases, infections, and malignancies [50]. During this pandemic, many studies have reported thrombotic and thromboembolic events among COVID-19 patients, wherein the patients had elevated d-dimer and fibrinogen levels and decreased antithrombin levels [51]. In patients with infection or sepsis that was diagnosed in the ED, a relationship was found between high levels of d-dimer and 28-day mortality [52]. In another study, it was shown that poor prognosis and fatality were more common in COVID-19 patients if the d-dimer levels were higher than 1 μg/mL [53]. Earlier studies have reported that the DFR has the potential to be a predictor for thrombotic/ thromboembolic events, stroke, and gastrointestinal stromal tumors [16–19]. The theory about the coagulation cascade is that when the system is activated to form fibrin in the pulmonary vasculature, fibrinogen is degraded into products, such as D-dimer, leading these products to be elevated in the bloodstream [54]. This theory may be applicable in COVID-19, without complications that may influence the DFR (as an acute-phase reactant) on admission to the ED. It was found herein that patients with a high FDR had poor prognosis. According to the results of the multivariate analysis, the FDR can be used as a good parameter for predicting severity in COVID-19 patients.

The NLP score was calculated for the COVID-19 patients, and the results showed that, if the score was higher than 6, the risk for progression to a severe disease was increased [6]. In terms of the NLP, the results obtained were similar to those of previous studies in the severe disease group. In this respect, the NLP score may be one of the important new parameters in terms of showing poor prognosis in COVID-19 patients. However, it was not found it as an independent risk factor in the current study. Among the COVID-19 patients, those who had a NLP score higher than 6 required more attention in terms of progression to severe disease. In this study, the specificity of a NLP score greater than 6 in predicting severe disease was 86.7%.

The SII is a tool that uses and combines 3 separate factors, comprising the neutrophil, lymphocyte, and platelet counts. In some studies, this parameter was used for the prediction of recurrency and survival in many solid tumors as a systemic inflammatory indicator [55,56]. In a recent study that was performed during the COVID-19 pandemic, it was reported that there was a positive correlation between the SII and the severity of the disease. In the current study, the calculated SII values were significantly higher in the severe disease group. The SII may be used as a new parameter with prognostic value in COVID-19 patients.

The diagnosis of COVID-19 is mainly confirmed with RT-PCR assays, depending on the detection of the SARS-CoV-2 virus [57], on the other hand for probable COVID-19 patients, primarily clinical, radiographic, and epidemiological features have importance on the initial medical examination [58]. Significant results were obtained when comparing the confirmed and probable groups of COVID-19 patients. Dyspnea was significantly higher in the probable patient group than in the confirmed patient group. This result was not surprising considering that one of the inclusion criteria in the probable patient group was radiological examination. In addition, while the patients in the probable group were older, smoking and comorbidities were most frequent in this group. Another interesting result in this study was that the need for intensive care was higher in the probable group. This may have been due to the fact that smoking and comorbidities were more frequent in this group, and the patients in this group were older.

The values of 8 combinations of inflammatory markers in patients with COVID-19 were calculated, and the predicted values of these 8 ratios were compared in the ROC analysis. The AUC values for the DFR were the highest among the 8 combinations of inflammatory markers. The NLR and SII were other inflammatory markers that had higher AUC values after the DFR.

Considering both the ROC analysis and the multivariate analysis together, the DFR and NLR are one step ahead in terms of predicting disease severity when compared to other hematological markers. CRP and IL-6 are inflammation-related biomarkers that have moderate-to-high correlation with the NLR, and these biomarkers are also associated with the unfavorable aspects of COVID-19 and duration of hospitalization [59,60]. Thus, for COVID-19, research has mostly been aimed at the NLR, to determine if it can be used as a valuable indicator to be able to make the decision to target the immune system [60]. It is difficult to routinely apply IL-6 and other cytokines in EDs or state hospitals, as they are not easily accessible and are expensive. IN addition to the increase in the DFR and NLR in inflammation, the fact that the NLR is correlated with cytokines, such as IL-6, stands out in the routine use of these combinations.

## 6. Conclusion

The NLR and DFR provide important prognostic information for decision making in severe patients with COVID-19. A high NLR, combined with the DFR, may be a better predictor of COVID-19 than other routinely used parameters in EDs. All patients with severe COVID-19 should be screened for hyperinflammation using the DFR and NLR to reduce mortality. The findings herein indicated that the parameters that they enhance from the complete blood count, which is a simple laboratory test, can help to identify and classify COVID-19 patients into non-severe to severe groups. Combining these parameters with the epidemiological data may be useful for the misdiagnosis or nondiagnosis of COVID-19.

## Figures and Tables

**Figure:**
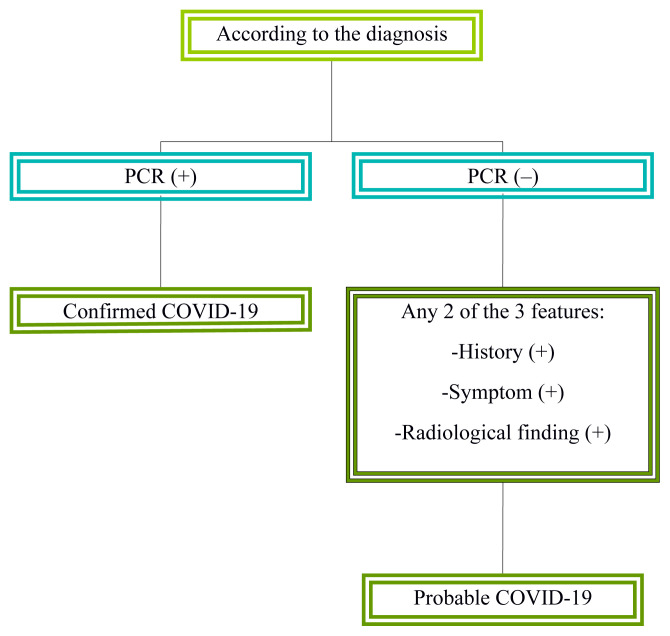
Diagram of distinction COVID-19 patients as confirmed and probable.

**Table 1:** Evaluation of the confirmed and probable patients according to clinical status, demographics, past-history, and laboratory parameters other than hematological parameters.

Characteristics or findings	All patients n: 750	Confirmed diagnosis n: 388	Probable disease n: 362	*P-value
Male sex, no. (%)	442 (58.9)	219 (56.4)	223 (61.6)	0.151
Median age, (IQR) years	49 (28)	45 (25)	55 (27)	<0.0001
Cough, no. (%)	396 (52.8)	227 (58.5)	169 (46.7)	0.001
Fever, no. (%)	284 (37.9)	179 (46.1)	105 (29.0)	<0.0001
Dyspnea, no. (%)	219 (29.2)	87 (22.4)	132 (36.5)	<0.0001
Headache, no. (%)	69 (9.2)	41 (10.6)	28 (7.7)	0.180
Nausea, no. (%)	47 (6.3)	29 (7.5)	18 (5)	0.207
Myalgia, no. (%)	166 (22.1)	109 (28.1)	57 (15.7)	<0.0001
Diarrhea, no. (%)	43 (5.7)	24 (6.2)	19 (5.2)	0.693
Back pain, no. (%)	3 (0.4)	2 (0.5)	1 (0.3)	1.000
Anosmia, no. (%)	37 (4.9)	26 (6.7)	11 (3)	0.032
Ageusia, no. (%)	33 (4.4)	243 (6.2)	9 (2.5)	0.022
Abdominal pain, no. (%)	11 (1.5)	2 (0.5)	9 (2.5)	0.052
Arthralgia, no. (%)	25 (3.3)	19 (4.9)	6 (1.7)	0.023
Smoking (smoker and nonsmoker), no. (%)	66 (29.5)	21 (20.6)	45 (36.9)	0.008
Any comorbidity, no. (%)	307 (40.9)	119 (30.7)	188 (51.9)	<0.0001
Hypertension, no. (%)	197 (26.3)	74 (19.1)	123 (34)	<0.0001
Diabetes, no. (%)	129 (17.2)	51 (13.1)	78 (21.5)	0.002
Asthma, no. (%)	42 (5.6)	16 (4.1)	26 (7.2)	0.097
Obesity, no. (%)	4 (0.5)	4 (1.0)	0 (0)	0.125
Coronary heart disease, no. (%)	111 (14.8)	31 (8.0)	80 (22.1)	<0.0001
Renal disease, no. (%)	37 (4.9)	6 (1.5)	31 (8.6)	<0.0001
COPD, no. (%)	37 (4.9)	9 (2.3)	28 (7.7)	0.001
Intensive care unit, no. (%)	119 (15.9)	44 (11.3)	75 (20.7)	<0.0001
Deceased, no. (%)	728 (97.1)	13 (3.4)	9 (2.5)	<0.0001
Creatin (mg/dL)	0.81 (0.29)	0.81 (0.27)	0.84 (0.33)	0.046
Lactate dehydrogenase (U/L)	225 (95.5)	218.5 (87.5)	236 (115)	0.002
C-reactive protein (g/L)	0.01 (0.04)	0.01 (0.02)	0.01 (0.05)	0.001
ESR (mm/h)	22 (36)	18 (31)	24 (42.25)	0.126
Ferritin concentration (μg/L)	127.5 (212.75)	122 (244.5)	134 (207.5)	0.357

COPD: chronic obstructive pulmonary disease, ESR: erythrocyte sedimentation rate.

* Statistical evaluations were made between probable and confirmed COVID-19 patients.

All laboratory parameters were calculated as the median (IQR).

**Table 2:** Evaluation of the hematological parameters between the confirmed and probable patients at the time of admission.

Parameters	All patients n: 750	Confirmed diagnosis n: 388	Probable disease n: 362	*P-value
WBC (10^9/L)	6.14 (3.43)	5.15 (2.48)	7.1 (3.9)	<0.0001
Neutrophil count (10^9/L)	3.90 (2.60)	3.30 (1.97)	4.66 (3.38)	<0.0001
Lymphocyte count (10^9/L)	1.38 (0.92)	1.25 (0.71)	1.5 (1.12)	<0.0001
Monocyte count (10^9/L)	0.4 (0.22)	0.35 (0.20)	0.43 (0.29)	<0.0001
Hemoglobin (g/dL)	13.6 (2.5)	13.8 (2.3)	13.4 (2.63)	0.031
Mpv (fL)	8.10 (1.35)	8.2 (1.2)	7.9 (1.3)	<0.0001
Plateletcrit (%)	0.2 (0.12)	0.18 (0.09)	0.22 (0.19)	<0.0001
Platelet (10^9/L)	231 (107)	209 (97.75)	249.5 (114.5)	<0.0001
NLR	2.65 (2.69)	2.45 (2.24)	2.98 (3.42)	<0.0001
PLR	166 (119.62)	167.04 (116.15)	163.10 (122)	0.388
LMR	3.67 (2.68)	3.66 (2.57)	3.70 (2.84)	0.900
MPVLR	5.83 (4.49)	6.63 (4.08)	4.61 (4.25)	<0.0001
MPVPR	0.03 (0.03)	0.04 (0.02)	0.03 (0.02)	<0.0001
Fibrinogen concentration (g/L)	3.5 (1.7)	3.36 (1.54)	3.8 (1.98)	<0.0001
D-dimer (mg/L)	0.5 (0.78)	0.43 (0.54)	0.61 (1.07)	<0.0001
DFR	0.14 (0.18)	0.14 (0.14)	0.15 (0.26)	0.002
SII	615.91 (759.52)	516.39 (557.09)	752.44 (982.55)	<0.0001
NLP score	4 (4)	4 (4)	4 (4)	0.002

WBC: white blood cell count, MPV: mean platelet volume, NLR: neutrophil/lymphocyte ratio, PLR: platelet/lymphocyte ratio, LMR: lymphocyte/monocyte ratio, MPVLR: MPV/lymphocyte ratio, MPVPR: MPV/platelet ratio, DFR: D-dimer/fibrinogen ratio, SII: systemic immune-inflammation index, N/LP: neutrophil/lymphocyte-platelet.

* Statistical evaluation were made between confirmed and probable patients.

All laboratory parameters were calculated as median (IQR).

**Table 3:** Evaluation of the hematological parameters among the confirmed, probable, and all patients according to the severity of the disease at the time of admission.

Parameters	All patients			Probable disease			Confirmed diagnosis		
	Nonsevere n: 631	Severe n: 119	P-value	Nonsevere n: 287	Severe n: 75	P-value	Nonsevere n: 344	Severe n: 44	P-value
Male sex	371 (58.8)	71 (59.7)	0.86	179 (62.4)	44 (58.7)	0.55	192 (55.8)	27 (61.4)	0.59
Age, years	46 (25)	67 (18)	<0.0001	50 (24)	67 (20)	<0.0001	42 (23)	67.5 (13.75)	<0.0001
Any comorbidity	224 (35.5)	83 (69.7)	<0.0001	131 (45.6)	57 (76)	<0.0001	93 (27)	26 (59.1)	<0.0001
WBC (10^9/L)	5.86 (3.08)	7.70 (5.42)	<0.0001	6.98 (3.60)	8.11 (5.9)	0.008	5.1 (2.25)	6.52 (3.97)	<0.0001
Neutrophil count (10^9/L)	3.62 (2.28)	5.42 (4.61)	<0.0001	4.47 (2.56)	6.12 (5.35)	<0.0001	3.18 (1.77)	4.78 (3.86)	<0.0001
Lymphocyte count (10^9/L)	1.42 (0.96)	1.08 (0.67)	<0.0001	1.65 (1.09)	1.15 (0.76)	<0.0001	1.30 (0.72)	1.00 (0.53)	<0.0001
Monocyte count (10^9/L)	0.39 (0.21)	0.41 (0.3)	0.122	0.42 (0.26)	0.47 (0.34)	0.484	0.35 (0.21)	0.40 (0.22)	0.407
Hemoglobin (g/dL)	13.8 (2.3)	12.1 (2.8)	<0.0001	13.7 (2.4)	12 (2.9)	<0.0001	13.9 (2.2)	12.3 (2.9)	<0.0001
Mpv (fL)	8 (1.3)	8.3 (1.7)	0.031	7.9 (1.33)	8.1 (1.4)	0.271	8.2 (1.2)	8.6 (1.75)	0.003
Plateletcrit (%)	0.2 (0.1)	0.24 (24.24)	0.008	0.22 (0.18)	0.24 (0.22)	0.708	0.18 (0.09)	0.23 (31.93)	0.02
Platelet (10^9/L)	230 (103)	235 (178)	0.321	249 (108)	254 (173)	0.642	210 (96)	199.5 (127)	0.95
NLR	2.43 (2.05)	5.43 (5.36)	<0.0001	2.57 (2.45)	5.59 (5.19)	<0.0001	2.34 (1.83)	5.05 (5.72)	<0.0001
PLR	157.82 (104.95)	216.5 (237.38)	<0.0001	151.18 (105.14)	206.22 (240.21)	<0.0001	160.31 (106.74)	241.2 (233.81)	<0.0001
LMR	3.88 (2.72)	2.59 (2.13)	<0.0001	4.05 (2.85)	2.67 (2.22)	<0.0001	3.74 (2.55)	2.52 (1.99)	<0.0001
MPVLR	5.62 (4.05)	7.33 (6.1)	<0.0001	4.42 (3.79)	6.17 (6.04)	0.001	6.42 (4.01)	8.84 (5.5)	<0.0001
MPVPR	0.03 (0.02)	0.03 (0.03)	0.642	0.03 (0.02)	0.03 (0.02)	0.775	0.04 (0.02)	0.04 (0.03)	0.453
Fibrinogen concentration (g/L)	3.39 (1.6)	4.37 (2.65)	<0.0001	3.6 (1.78)	4.48 (3.35)	<0.0001	3.22 (1.47)	4.1 (1.96)	<0.0001
D-dimer (mg/L)	0.43 (0.54)	1.60 (2.84)	<0.0001	0.48 (0.72)	1.82 (2.72)	<0.0001	0.40 (0.43)	1.17 (2.97)	<0.0001
DFR	0.13 (0.13)	0.37 (0.81)	<0.0001	0.13 (0.18)	0.38 (0.88)	<0.0001	0.12 (0.11)	0.32 (0.83)	<0.0001
SII	550.48 (598.67)	1.358.94 (1.887.2)	<0.0001	643.07 (729.72)	1.455.50 (2.030.06)	<0.0001	485.63 (451.77)	1.161.77 (1.829.86)	<0.0001
NLP score	4 (4)	4 (4)	<0.0001	4 (4)	4 (4)	<0.0001	4 (4)	4 (7)	<0.0001

Abbreviations: WBC; White blood cell count, MPV; Mean platelet volume, NLR; Neutrophil/lymphocyte ratio, PLR; Platelet/lymphocyte ratio, LMR; Lymphocyte/monocyte ratio, MPVLR; MPV/lymphocyte ratio, MPVPR; MPV/platelet ratio, DFR; D-dimer/fibrinogen ratio, SII; Systemic immun-inflammation index, NLP score; Neutrophil-lymphocyte-platelet score

All laboratory parameters have been calculated as median (IQR).

**Table 4:** Evaluation of the hematological parameters of all of the patients according to the severity of disease with multivariate logistic regression analyses at the time of admission.

Parameters	All patients		
	OR	95% CI	P-value
Male sex	1.213	0.748–1.967	0.434
Age, years	1.071	1.055–1.088	<0.0001
Any comorbidity	1.304	0.763–2.227	0.332
Plateletcrit (%)	1.015	1.000–1.030	0.049
NLR	1.076	1.028–1.127	0.002
PLR	1.002	1.000–1.004	0.027
LMR	0.995	0.960–1.031	0.776
MPVLR	0.989	0.930–1.051	0.718
MPVPR	1.005	0.778–1.299	0.970
DFR	1.206	1.049–1.387	0.009
SII	1.000	1.000–1.000	0.556
NLP score	1.044	0.948–1.149	0.383

Abbreviations: WBC; White blood cell count, MPV; Mean platelet volume, NLR; Neutrophil/lymphocyte ratio, PLR; Platelet/ lymphocyte ratio, LMR; Lymphocyte/monocyte ratio, MPVLR; MPV/lymphocyte ratio, MPVPR; MPV/platelet ratio, DFR; D-dimer/fibrinogen ratio, SII; Systemic immun-inflammation index, NLP score; Neutrophil-lymphocyte-platelet score.

**Table 5:** AUC and optimal thresholds of each independent risk or protection factors for the hematological parameters of all of the patients according to the severity of the disease.

Indicators	AUC	P-value	Optimal threshold	Sensitivity	Specificity	Youden index
WBC (10^9/L)	0.653	<0.0001	>7.69	50.42	74.17	0.246
Neutrophil count (10^9/L)	0.702	<0.0001	>4.7	62.18	70.05	0.322
Lymphocyte count (10^9/L)	0.664	<0.0001	≤1.11	57.98	69.05	0.270
Monocyte count (10^9/L)	0.545	0.137	>0.39	60.5	51.43	0.119
Hemoglobin (g/dL)	0.708	<0.0001	≤12.8	66.39	71.16	0.375
Mpv (fL)	0.562	0.043	>8.9	30.25	82.22	0.125
Plateletcrit (%)	0.577	0.013	>0.28	43.70	78.73	0.224
Platelet (10^9/L)	0.529	0.385	>335	29.41	87.96	0.174
NLR	0.750	<0.0001	>3.59	70.59	71.43	0.420
PLR	0.661	<0.0001	>279.07	42.02	86.67	0.287
LMR	0.689	<0.0001	≤3.08	65.55	65.56	0.311
MPVLR	0.620	<0.0001	>6.91	57.98	66.35	0.243
MPVPR	0.513	0.676	≤0.02	34.45	76.83	0.113
Fibrinogen concentration (g/L)	0.684	<0.0001	>4.34	50.42	78.13	0.286
DFR	0.767	<0.0001	>0.22	67.23	75.75	0.430
SII	0.740	<0.0001	>998.28	63.87	76.35	0.402
NLP score	0.665	<0.0001	6	32.8	86.7	0.246

Abbreviations: WBC; White blood cell count, MPV; Mean platelet volume, NLR; Neutrophil/lymphocyte ratio, PLR; Platelet/lymphocyte ratio, LMR; Lymphocyte/monocyte ratio, MPVLR; MPV/Lymphocyte ratio, MPVPR; MPV/platelet ratio, DFR; D-dimer/fibrinogen ratio, SII; Systemic immun-inflammation index, NLP score; Neutrophil-lymphocyte-platelet score.

## Footnotes

1 World Health Organization (2020). Clinical Management of Severe Acute Respiratory Infection When Novel Coronavirus (2019-nCoV) Infection is Probable. Interim Guidance, 13 March 2020 [Online]. Website: https://apps.who.int/iris/handle/10665/330854 [accessed 14 April 2020].

2 Republic of Turkey Ministry of Health Directorate General of Public Health (2020). COVID-19 (SARS-CoV-2 Infection) guide (in Turkish) [online]. Website: https://covid19bilgi.saglik.gov.tr/depo/rehberler/COVID-19_Rehberi.pdf [accessed 14 April 2020].
